# Robotic-assisted surgery in Egypt: national insights into awareness, knowledge, and perceptions among surgeons and patients

**DOI:** 10.1007/s11701-025-02942-w

**Published:** 2025-11-21

**Authors:** Mohamed F. Srour, Ahmed H. Shoaib, Hazim Alkousheh, Karim K. Eladawy, Mohamed Alayat, Ahmed Abdelhameed, Osama Alhaddad, Seif M. Elsadik, Ezzeldin Ahmed Abdelaty, Mohamed Sloma, Nada Rady, Mohammad A. Abd-erRazik

**Affiliations:** 1 Faculty of Medicine, Menoufia University, Menoufia, Egypt; 2 Faculty of Medicine, Alexandria University, Alexandria, Egypt; 3 Faculty of Medicine, The Hashemite University, Zarqa, Jordan; 4 Faculty of Medicine, Ain Shams University, Cairo, Egypt; 5 Faculty of Medicine, Al-Azhar University, Cairo, Egypt; 6 Faculty of Medicine, October 6 University, Giza, Egypt; 7 Faculty of Medicine, Al-Azhar University, Damietta, Egypt; 8 General Surgery, Faculty of Medicine, Ain Shams University, Cairo, Egypt

**Keywords:** Robotic-assisted surgery, Awareness, Perceptions, Surgeons, Patients, Egypt

## Abstract

The integration of robotic-assisted surgery (RAS) in low- and middle-income countries (LMIC) is hindered by significant perception gaps among key stakeholders. This study evaluated the awareness, knowledge, and perceptions of RAS in Egypt to identify barriers to its adoption. A national, multi-center, cross-sectional study was conducted across ten Egyptian university hospitals (Nov 2024–Apr 2025). Validated questionnaires were administered to three cohorts: general surgical patients (*n* = 539), surgeons (*n* = 486), and RAS-experienced patients (*n* = 32). Multivariable logistic regression identified predictors of patient preference and surgeon support for RAS. A stark perception gap was observed. Surgeon awareness was high (90.7%), but knowledge was limited; only 56.8% knew RAS was available in Egypt. Despite this, 80.0% supported national implementation, which was independently predicted by awareness of local availability (aOR 6.35, 95% CI 3.25–12.42) and correct technical understanding (aOR 2.49, 1.48–4.18). Conversely, patient awareness was low (30.4%), with high prevalence of fears regarding malfunction (63.3%). Only 20.0% preferred RAS. Patient preference was associated with the absence of fears (aOR 0.08, 0.04–0.16) and belief in improved outcomes (aOR 4.08, 2.10–7.94). RAS-experienced patients, informed primarily by surgeons (90.6%), reported high satisfaction (84.4% “Very Good”/“Excellent”) and were strong advocates (90.6% would recommend it). RAS adoption in Egypt appears to be influenced mainly by modifiable perceptual barriers rather than demographics. Surgeons are generally supportive but under-informed, while patient hesitancy seems driven by fear that can lessen after direct exposure and education. National strategies should prioritize targeted education and the use of positive patient experiences to help address these gaps.

**Supplementary Information:**

The online version contains supplementary material available at 10.1007/s11701-025-02942-w.

## Introduction

Robotic-assisted surgery (RAS) represents a paradigm shift in minimally invasive care, offering potential enhancements in surgical precision, dexterity, and three-dimensional visualization [1–4]. Since the introduction of the da Vinci Surgical System, RAS utilization has expanded globally, now encompassing millions of procedures across urologic, gynecologic, general, and cardiothoracic specialties [5]. A growing body of evidence documents its clinical benefits, including reduced intraoperative blood loss, shorter hospital stays, and faster patient recovery times, although debates regarding cost-effectiveness and long-term outcomes persist [1–4].

The global diffusion of RAS, however, follows a markedly inequitable pattern. While high-income countries have rapidly integrated this technology into clinical practice, its adoption in low- and middle-income countries (LMICs) remains limited and uneven [6–8]. The conventional narrative predominantly focuses on prohibitive acquisition costs and maintenance as the primary barriers to initial implementation [8]. Crucially, emerging evidence suggests that beyond these financial hurdles, the sustainable adoption and scaling of complex medical technologies is critically dependent on the acceptance, understanding, and perceptions of its key stakeholders: surgeons and patients [9, 10]. Misconceptions about intraoperative autonomy, fears of system malfunction, and a simple lack of awareness can stifle demand and hinder widespread utilization, even in settings where the financial resources for procurement exist [9, 11].

The Middle East and North Africa (MENA) region exemplifies this contrast. While high-income Gulf nations like Saudi Arabia and Kuwait have the financial capacity for initial implementation and have established advanced RAS programs, studies from these very countries reveal significant perception gaps and knowledge deficits among both the public and medical professionals [12–14]. This suggests that financial investment is a necessary but insufficient condition for full integration into a healthcare ecosystem; attitudinal barriers can persist and limit impact long after the technology is acquired. Egypt, the Arab world’s most populous nation, represents a critical and understudied case. It introduced RAS significantly later than its regional peers, with the first procedures performed in 2021 [15]. The initial applications have been reported in complex procedures [16], confirming the technology’s presence but also highlighting its nascent stage and limited dissemination across the healthcare system. This recent inception presents a unique opportunity to study the initial phases of technological diffusion in a resource-constrained environment, where stakeholder perceptions are poised to play a decisive role in its future trajectory.

To our knowledge, this is the first national, multi-center study assessing awareness, knowledge, and perceptions of RAS in Egypt, and one of the largest in the MENA region. Understanding these factors is not merely academic; it is an essential prerequisite for designing effective, data-driven implementation strategies that ensure equitable and sustainable access to surgical innovation. To address this critical knowledge gap, we conducted a national, multi-center study simultaneously surveying three crucial cohorts: general surgical patients, surgeons of all specialties and seniority, and patients with firsthand RAS experience. Our primary aims were to: (1) quantify the levels of awareness and knowledge of RAS among surgeons and patients; (2) identify prevailing perceptions, misconceptions, and fears within each group; (3) evaluate the procedural experiences and satisfaction of patients who underwent RAS; and (4) determine the independent factors predicting patient preference for RAS and surgeon support for its national implementation.

## Materials and methods

### Study design and setting

We conducted a national, multi-center, cross-sectional observational study across 10 geographically diverse governmental university hospitals in Egypt. The study was designed to assess awareness, knowledge, perceptions, and experiences related to robotic-assisted surgery (RAS) among three distinct groups: surgeons, general surgical patients, and patients with prior RAS experience. Data collection occurred from November 2024 to April 2025. A pilot study at two centers (El-Hussein and Ain Shams University Hospitals) was used to develop and validate the study questionnaires, which demonstrated excellent content validity (I-CVI = 1.0; S-CVI/Ave = 1.0), clarity, and comprehensibility. The data used in the pilot study were not included in this study.

### Participants

Eligible participants were adults (≥ 18 years) recruited from three cohorts: (1) surgeons of all specialties and training levels (junior/senior residents, consultants); (2) patients attending surgical outpatient clinics; and (3) patients who had undergone RAS in Egypt. Exclusion criteria included presentation with surgical emergencies or cognitive/communication impairments that could compromise response validity.

General surgical patients were recruited via convenience sampling from various surgical outpatient clinics’ waiting areas across the ten participating university hospitals. Trained data collectors approached consecutive adult patients present in the waiting rooms during the data collection period and invited voluntary participation; questionnaires were completed in-person while patients awaited clinic appointments. We did not restrict recruitment to pre-operative patients only, and thus the sample includes patients attending clinics for both pre-operative assessment and routine follow-up, reflecting general surgical clinic attendees rather than a disease-specific subgroup.

RAS-experienced patients were identified from Robotic Surgery Unit records at the Ain-Shams Specialized University Hospital. All 50 eligible patients were contacted consecutively by phone or WhatsApp and asked to complete the questionnaire via Google Form or phone interview; 32 responded (response rate 64%). Surgeons were recruited in-person via convenience sampling from various surgical departments: data collectors visited various surgical departments and invited on-duty surgeons and those present in the departments to complete the questionnaire face-to-face.

### Study instruments and validation

Three structured questionnaires were developed: one for surgeons (English, 25 items), one for general patients (Arabic, 21 items), and one for RAS patients (Arabic, 26 items). Instruments were adapted from existing literature [12, 13, 17] and refined through expert review for face validity, cultural sensitivity, and the pilot validation process involving cognitive interviews. The questionnaires assessed demographics, digital literacy, and RAS-specific awareness, knowledge, perceptions, and experiences. The full instruments are provided in Online Resources 1–3.

### Data collection procedures

Trained data collectors conducted interviews using printed or digital forms (Google Forms via tablets/smartphones). For RAS patients, interviews were conducted in-person, by phone, or online to accommodate accessibility. A list of all eligible RAS patients was compiled from surgical records at Ain-Shams Specialized University Hospital. We attempted to contact all 50 identified individuals consecutively; 32 completed the survey (response rate = 64%).

### Sample size calculation

Sample size was calculated for each group using the formula for proportions: n = (Z² * P(1-P))/d² [18], where Z = 1.96 (95% CI), *P* = 0.5 (maximum variability), and d = 0.05 (margin of error). Adjusting for a 10% non-response rate, target sample sizes were 333 surgeons, 348 surgical patients, and 96 RAS patients (total *n* = 777). We aimed to recruit over 800 participants to ensure robust enrollment across centers and enable precise subgroup analyses.

### Ethical considerations

This study adhered to STROBE guidelines and the Declaration of Helsinki. Ethical approval was obtained from the Research Ethics Committees (RECs) of Menoufia University (11/2024UROL1), Ain-Shams University (IRB 00006379), Al Azhar University (IRB 00012367), Benha University (MoHP 0018122017/Certificate 1017), and Alexandria University (IRB 00012098). Verbal informed consent was obtained from all participants. Participation was voluntary, anonymous, and confidential.

### Statistical analysis

Data were analyzed using Jamovi (v2.3.28) and RStudio (2023.03.1). Descriptive statistics are presented as frequencies (percentages) for categorical variables and as mean ± standard deviation (SD) or median [interquartile range] for continuous variables, based on their distribution. Group comparisons were performed using Chi-square, Fisher’s exact, t-tests, Mann-Whitney U, ANOVA, or Kruskal-Wallis tests as appropriate.

Multivariable logistic regression was employed to identify independent predictors of two primary outcomes: (1) patient preference for RAS (categorized as ‘Yes’ vs. ‘No/Not Sure’), and (2) surgeon support for national RAS implementation (‘Agree’ vs. ‘Disagree/Not Sure’). Variables with a univariate association of p < 0.1 were included in initial models. Final models were constructed using a backward stepwise selection process and are reported as adjusted odds ratios (aOR) with 95% confidence intervals (CI). The goodness-of-fit for the patient preference model was assessed, and its predictive accuracy was evaluated using Nagelkerke’s R². A two-sided p-value < 0.05 was considered statistically significant.

## Results

### Patient cohort: demographics, awareness, and determinants of preference for RAS

A total of 539 general surgical patients participated in the study. The cohort was predominantly male (64.2%) with a median age of 35 years (IQR 23); full demographic and technological characteristics are presented in Table 1.

**Table 1:** Demographic characteristics and technological experience of participants

	Patients (*N* = 539)	Surgeons (*N* = 486)	RAS-patients (*N* = 32)
**Age**			
Median (IQR)	35 (23)	29 (7)	45.5 (23.8)
**Gender**			
Female	193 (35.8%)	81 (16.7%)	21 (65.6%)
Male	346 (64.2%)	405 (83.3%)	11 (34.4%)
**Nationality**			
Egyptian	536 (99.4%)	481 (99%)	30 (93.8%)
Non-Egyptian	3 (0.6%)	5 (1%)	2 (6.2%)
**Speciality**			
General surgery	-	175 (36%)	-
Urology	-	42 (8.6%)	-
Obstetrics and Gynaecology	-	71 (14.6%)	-
Cardiothoracic surgery	-	15 (3.1%)	-
Orthopaedic surgery	-	44 (9.1%)	-
ENT	-	19 (3.9%)	-
Neurosurgery	-	26 (5.3%)	-
Paediatric surgery	-	32 (6.6%)	-
Vascular surgery	-	36 (7.4%)	-
Plastic surgery	-	20 (4.1%)	-
Ophthalmology	-	5 (1.0%)	-
Head and Neck Surgery	-	1 (0.2%)	-
**Employment level**			
Consultant	-	95 (19.5%)	-
Specialist/Assistant Lecturer	-	83 (17.1%)	-
Registrar/Senior Resident	-	138 (28.4%)	-
Assistant Registrar/Junior Resident	-	170 (35%)	-
**Level of education**			
Primary school	126 (23.4%)	-	5 (15.6%)
Secondary school	52 (9.6%)	-	1 (3.1%)
Diploma	137 (25.4%)	-	3 (9.4%)
Bachelor’s	212 (39.3%)	-	20 (62.5%)
Postgraduate studies	12 (2.2%)	-	3 (9.4%)
**Hours of computer use per day**			
Median (IQR)	3 (5)	3 (3)	4 (6)
**Level of comfort with the computer**			
Comfortable	241 (44.7%)		14 (43.8%)
Somewhat comfortable	184 (34.1%)		16 (50%)
Uncomfortable	114 (21.2%)		2 (6.2%)
**Level of computer experience**			
No experience	235 (43.6%)	-	4 (12.5%)
Some experience	242 (44.9%)	-	25 (78.1%)
Advanced experience	62 (11.5%)	-	3 (9.4%)
**Level of computer proficiency**			
None: Cannot use a computer		1 (0.2%)	
Beginner: Can perform basic tasks like starting the computer, browsing the internet, and using email		99 (20.4%)	
Intermediate: Can use productivity software (e.g., Word, Excel) and solve basic technical issues		283 (58.2%)	
Advanced: Can manage complex software, troubleshoot systems, and use advanced features of applications		94 (19.3%)	
Expert: Can write or customise software programs and manage IT infrastructure		9 (1.9%)	

Awareness of robotic-assisted surgery was strikingly low; only 164 patients (30.4%) had heard of RAS prior to the study. Among aware patients, the internet and social media were the most common sources of information (78.0%, Online Resource 4). Knowledge of RAS fundamentals was poor. Only 26.3% (*n* = 142) correctly identified the surgeon’s role in controlling the robotic arms, and 32.3% (*n* = 174) accurately recognized its similarity to laparoscopic surgery. Furthermore, only 12.1% (*n* = 65) were aware that RAS is available in Egypt.

Patient perceptions were defined by significant fears and limited recognition of benefits. Concerns were prominent, with 63.3% fearing intraoperative robot malfunction and 42.1% fearing the robot performing an incorrect procedure. While 59.4% recognized RAS as more expensive, fewer perceived its advantages: 33.0% believed it yields better results, 21.5% thought it safer, and 21.2% perceived it as less painful. Consequently, when asked their preference, only 20.0% (*n* = 108) of patients preferred RAS, while 53.4% (*n* = 288) declined and 26.5% (*n* = 143) were unsure. Further details in Table 2.

**Table 2:** Patient demographics, knowledge, awareness, and perceptions about robotic surgery compared by patients’ preference for having robotic surgery

	Yes (*N* = 108)	Not sure (*N* = 143)	No (*N* = 288)	Total (*N* = 539)	*p* value
**Age**					0.061^a^
Median (IQR)	35.5 (22)	35 (18)	37 (26.3)	35 (23)	
**Gender**					0.227^b^
Female	31 (28.7%)	54 (37.8%)	108 (37.5%)	193 (35.8%)	
Male	77 (71.3%)	89 (62.2%)	180 (62.5%)	346 (64.2%)	
**Level of education**					0.263^c^
Primary school	21 (19.4%)	35 (24.5%)	70 (24.3%)	126 (23.4%)	
Secondary school	16 (14.8%)	8 (5.6%)	28 (9.7%)	52 (9.6%)	
Diploma	28 (25.9%)	36 (25.2%)	73 (25.3%)	137 (25.4%)	
Bachelor’s	42 (38.9%)	58 (40.6%)	112 (38.9%)	212 (39.3%)	
Postgraduate studies	1 (0.9%)	6 (4.2%)	5 (1.7%)	12 (2.2%)	
**Hours of computer use per day**					0.095^a^
Median (IQR)	4 (4)	3 (4)	3 (5)	3 (5)	
**Level of comfort with the computer**					0.355^b^
Comfortable	55 (50.9%)	67 (46.9%)	119 (41.3%)	241 (44.7%)	
Somewhat comfortable	35 (32.4%)	49 (34.3%)	100 (34.7%)	184 (34.1%)	
Uncomfortable	18 (16.7%)	27 (18.9%)	69 (24.0%)	114 (21.2%)	
**Level of computer experience**					0.238^b^
Advanced experience	17 (15.7%)	11 (7.7%)	34 (11.8%)	62 (11.5%)	
Some experience	51 (47.2%)	68 (47.6%)	123 (42.7%)	242 (44.9%)	
No experience	40 (37%)	64 (44.8%)	131 (45.5%)	235 (43.6%)	
**Heard about robotic surgery**					< 0.001^b^
Yes	56 (51.9%)	51 (35.7%)	57 (19.8%)	164 (30.4%)	
No	52 (48.1%)	92 (64.3%)	231 (80.2%)	375 (69.6%)	
**Understanding of robotic surgery**					< 0.001^b^
Robot performs surgery, trained surgeon stands by	11 (10.2%)	16 (11.2%)	44 (15.3%)	71 (13.2%)	
Surgeon controls robotic arms and instruments (correct answer)	40 (37%)	41 (28.7%)	61 (21.2%)	142 (26.3%)	
Surgeon tells robot what to do, robot follows each command	23 (21.3%)	27 (18.9%)	40 (13.9%)	90 (16.7%)	
Surgeon does not present in the operating theatre; robot performs according to software	21 (19.4%)	7 (4.9%)	35 (12.2%)	63 (11.7%)	
I don’t know	13 (12%)	52 (36.4%)	108 (37.5%)	173 (32.1%)	
**Which type of surgery is robotic surgery most similar to?**					< 0.001^b^
Traditional open surgery	9 (8.3%)	4 (2.8%)	19 (6.6%)	32 (5.9%)	
Laparoscopic surgery	52 (48.1%)	48 (33.6%)	74 (25.7%)	174 (32.3%)	
Laser surgery	23 (21.3%)	31 (21.7%)	62 (21.5%)	116 (21.5%)	
I don’t know	24 (22.2%)	60 (42%)	133 (46.2%)	217 (40.3%)	
**Is robotic surgery available in Egypt?**					< 0.001^b^
Yes	24 (22.2%)	19 (13.3%)	22 (7.6%)	65 (12.1%)	
Not sure	47 (43.5%)	96 (67.1%)	141 (49%)	284 (52.7%)	
No	37 (34.3%)	28 (19.6%)	125 (43.4%)	190 (35.3%)	
**Perceptions of robotic surgery compared to non-robotic surgery (Multiple responses)**					
Safer	55 (50.9%)	27 (18.9%)	34 (11.8%)	116 (21.5%)	< 0.001^b^
Less painful	45 (41.7%)	28 (19.6%)	41 (14.2%)	114 (21.2%)	< 0.001^b^
Better results	72 (66.7%)	41 (28.7%)	65 (22.6%)	178 (33%)	< 0.001^b^
Faster	53 (49.1%)	68 (47.6%)	116 (40.3%)	237 (44%)	0.175^b^
More expensive	62 (57.4%)	88.0 (61.5%)	170 (59.0%)	320 (59.4%)	0.792^b^
None of the above	5 (4.6%)	12 (8.4%)	39 (13.5%)	56 (10.4%)	0.023^b^
**Fears regarding robotic surgery (Multiple responses)**					
Robot malfunctions during surgery	41 (38%)	96 (67.1%)	204 (70.8%)	341 (63.3%)	< 0.001^b^
Robot performs wrong procedure	21 (19.4%)	55 (38.5%)	151 (52.4%)	227 (42.1%)	< 0.001^b^
I don’t have fears regarding robotic surgery	62 (57.4%)	24 (16.8%)	20 (6.9%)	106 (19.7%)	< 0.001^b^
**Perceptions of surgeons trained in robotic surgery**					< 0.001^b^
More skilled	89 (82.4%)	81 (56.6%)	124 (43.1%)	294 (54.5%)	
Similar	11 (10.2%)	53 (37.1%)	88 (30.6%)	152 (28.2%)	
Less skilled	8 (7.4%)	9 (6.3%)	76 (26.4%)	93 (17.3%)	
**Perceptions of hospitals using robotic surgery**					< 0.001^b^
Better	96 (88.9%)	96 (67.1%)	170 (59%)	362 (67.2%)	
Similar	10 (9.3%)	39 (27.3%)	84 (29.2%)	133 (24.7%)	
Worse	2 (1.9%)	8 (5.6%)	34 (11.8%)	44 (8.2%)	
**Compared to standard laparoscopic surgery**,** robotic procedures cost**					0.233^c^
More	89 (82.4%)	120 (83.9%)	256 (88.9%)	465 (86.3%)	
Same	15 (13.9%)	16 (11.2%)	20 (6.9%)	51 (9.5%)	
Less	4 (3.7%)	7 (4.9%)	12 (4.2%)	23 (4.3%)	
**Robotic surgery can replace the currently used surgical procedures**					< 0.001^b^
Yes	67 (62%)	32 (22.4%)	66 (22.9%)	165 (30.6%)	
Not sure	15 (13.9%)	79 (55.2%)	72 (25%)	166 (30.8%)	
No	26 (24.1%)	32 (22.4%)	150 (52.1%)	208 (38.6%)	
**The use of robotics in surgeries can improve surgical outcomes**					< 0.001^b^
Yes	84 (77.8%)	50 (35%)	55 (19.1%)	189 (35.1%)	
Not sure	19 (17.6%)	85 (59.4%)	145 (50.3%)	249 (46.2%)	
No	5 (4.6%)	8 (5.6%)	88 (30.6%)	101 (18.7%)	

^a^ Kruskal-Wallis H test

^b^ Pearson’s Chi-squared test

^c^ Fisher’s Exact test

### Factors associated with patient preference for robotic-assisted surgery

A multivariable logistic regression analysis revealed that modifiable perceptions and awareness were the dominant drivers of patient choice, outweighing demographic factors (Table 3). The model explained a substantial proportion of the variance in patient preference (Nagelkerke R² = 0.460).

**Table 3:** Multivariable logistic regression analysis of factors associated with patient preference for Robotic-Assisted surgery

Predictor	Adjusted Odds Ratio (aOR)	95% CI	*p*-value
Education level	0.37	0.17–0.78	0.009
Hours of computer use per day	1.12	1.01–1.23	0.031
Heard about RAS	2.15	1.10–4.17	0.024
Perceives RAS as safer	2.23	1.18–4.24	0.014
Perceives RAS as less painful	3.05	1.56–5.95	0.001
Perceives RAS as having better results	3.77	2.00–7.09	< 0.001
Has fears regarding RAS	0.08	0.04–0.16	< 0.001
Perceives RAS surgeons as more skilled	2.65	1.36–5.18	0.004
Believes robotics improves surgical outcomes	4.08	2.10–7.94	< 0.001

CI = confidence interval

The model is adjusted for all variables listed in the table, as well as age and gender

*The outcome variable is preference for robotic-assisted surgery (Yes vs. No/Not Sure). Nagelkerke R² = 0.460.*

The most powerful predictor was the absence of fears regarding RAS. Patients who expressed any fears were less likely to prefer it than those who reported no fears (aOR 0.08, 95% CI 0.04–0.16, *p* < 0.001).

Conversely, positive perceptions were strong positive drivers. Patients who believed robotics could improve surgical outcomes were over four times more likely to prefer it (aOR 4.08, 95% CI 2.10–7.94, *p* < 0.001). Those who perceived RAS as providing better results were nearly four times more likely to prefer it (aOR 3.77, 95% CI 2.00–7.09, *p* < 0.001), and those who perceived it as less painful were three times more likely (aOR 3.05, 95% CI 1.56–5.95, *p* = 0.001).

Prior awareness of RAS also doubled the odds of preference (aOR 2.15, 95% CI 1.10–4.17, *p* = 0.024). Notably, patients with a bachelor’s degree or higher were less likely to prefer RAS than those with lower educational attainment (aOR 0.37, 95% CI 0.17–0.78, *p* = 0.009).

### Surgeon cohort: demographics, knowledge, and determinants of support for RAS implementation

A total of 486 surgeons participated. The cohort was predominantly male (83.3%) and young, with a median age of 29 years (IQR 7), and composed primarily of junior and senior residents (63.4% residents). General surgery (36.0%), obstetrics and gynaecology (14.6%), and orthopaedics (9.1%) were the most represented specialties; full demographics are presented in Table 1.

Awareness of RAS was nearly ubiquitous among surgeons (90.7%, *n* = 441), with medical conferences, colleagues, and the internet/social media being the primary sources of information (Online Resource 4). However, in-depth knowledge was variable. While 71.2% (*n* = 346) correctly understood that the surgeon controls the robotic arms, only 56.2% (*n* = 273) believed the surgeon has complete control during the procedure. Awareness of RAS availability in Egypt was also limited; only 56.8% (*n* = 276) correctly knew it was available.

**Fig. 1:**
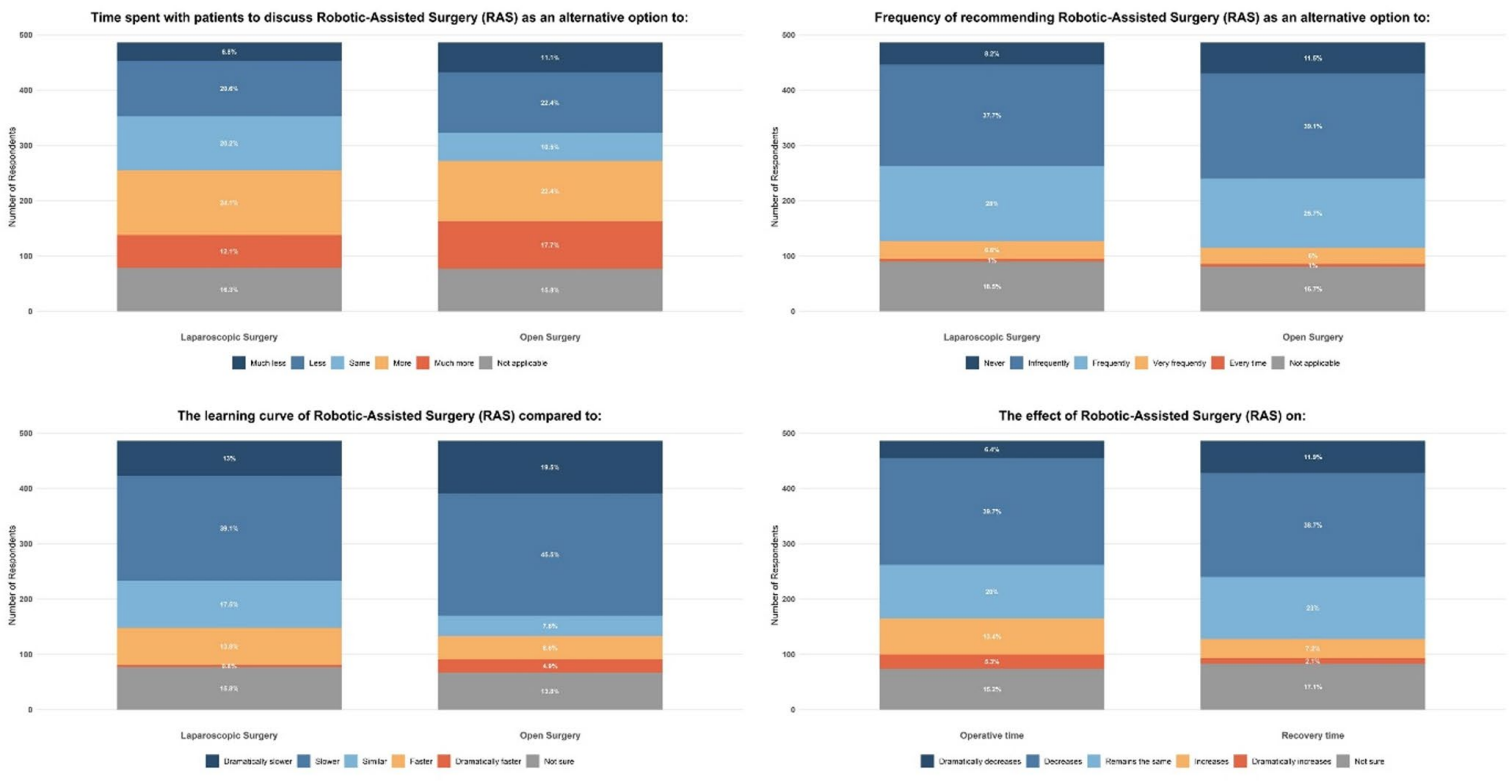
Surgeon perceptions of Robotic-Assisted Surgery (RAS) compared to laparoscopic and open surgery across key domains including time required for patient discussion, learning curve, frequency of recommendation, perceived effect on operative time, and cost

Despite these knowledge gaps, a strong majority of surgeons (80.0%, *n* = 389) supported introducing RAS into the healthcare system. The primary reasons for support were the potential for improved 3D visualization and magnification (68.6%), more precise intraoperative performance (63.0%), and fewer complications (49.9%). Among the minority who opposed introduction (7.6%, *n* = 37), the leading concerns were high cost (62.2%), limited availability (59.5%), and the perception that laparoscopic surgery offers similar benefits (45.9%) (Online Resource 4). Surgeons held mixed perceptions of RAS’s practicality. While advantages for patient recovery (50.6%) and operative time (46.1%) were recognized, these were counterbalanced by a perceived steep learning curve and significantly higher cost (89.1% vs. laparoscopy). Consequently, surgeons anticipated spending more time discussing RAS with patients and were hesitant to recommend it frequently as an alternative to open (32.7%) or laparoscopic (35.6%) surgery (Fig. 1).

### Impact of institutional RAS availability on surgeon perceptions

Surgeons at hospitals with RAS available (*n* = 141, 29.0%) were significantly more informed and supportive than those without access (*n* = 345, 71.0%). They were more likely to be aware of its national availability (94.3% vs. 41.4%, *p* < 0.001), to correctly understand the surgeon’s role in controlling the robot (83.0% vs. 66.4%, *p* = 0.001), and to support its introduction (92.9% vs. 74.8%, *p* < 0.001). They were also less likely to cite difficult training as a major limitation (13.6% vs. 44.7%, *p* < 0.001). Financial burden was a prevalent concern in both groups, though more so among those without local access (72.9% vs. 89.4%, *p* < 0.001). Further information in Table 4.

**Table 4:** Surgeons’ perceptions about robotic surgery compared by the availability of RAS in the same hospital as the surgeon

	Available (*N* = 141, 29%)	Not available (*N* = 345, 71%)	Total (*N* = 486)	*p* value*
**Heard about robotic surgery**				< 0.001^a^
Yes	141 (100%)	300 (87%)	441 (90.7%)	
No	0	45 (13%)	45 (9.3%)	
**Understanding of robotic surgery**				0.001^a^
The robot performs the surgery while a trained surgeon stands by and makes sure the robot does things correctly	11 (7.8%)	30 (8.7%)	41 (8.4%)	
The surgeon controls the robotic arms and instruments, but the surgeon does all of the operating	117 (83%)	229 (66.4%)	346 (71.2%)	
The surgeon programs the moves the robot should make, then the robot does the surgery	9 (6.4%)	59 (17.1%)	68 (14.0%)	
The surgeon tells the robot what to do, and the robot then follows each command	4 (2.8%)	27 (7.8%)	31 (6.4%)	
**Level of surgeon control during robotic surgery**				< 0.001^b^
Complete control	100 (70.9%)	173 (50.1%)	273 (56.2%)	
Major control	28 (19.9%)	138 (40%)	166 (34.2%)	
Minimal control	5 (3.5%)	19 (5.5%)	24 (4.9%)	
No control	1 (0.7%)	0	1 (0.2%)	
Not sure	7 (5%)	15 (4.3%)	22 (4.5%)	
**Is robotic-assisted surgery currently available in Egypt?**				< 0.001^a^
Yes	133 (94.3%)	143 (41.4%)	276 (56.8%)	
No	2 (1.4%)	84 (24.3%)	86 (17.7%)	
I don’t know	6 (4.3%)	118 (34.2%)	124 (25.5%)	
**What about introducing robotic surgery into the healthcare system**				< 0.001^a^
Agree	131 (92.9%)	258 (74.8%)	389 (80%)	
Disagree	1 (0.7%)	36 (10.4%)	37 (7.6%)	
Not sure	9 (6.4%)	51 (14.8%)	60 (12.3%)	

^a^ Pearson’s Chi-squared test

^b^ Fisher’s exact test

### Factors associated with surgeon support for RAS implementation

Multivariable logistic regression identified key determinants of surgeon support (Online Resource 4). The strongest predictor was knowledge that RAS is available in Egypt; surgeons with this awareness were over six times more likely to support its introduction (aOR 6.35, 95% CI 3.25–12.42, *p* < 0.001). A correct technical understanding of robotic surgery also more than doubled the odds of support (aOR 2.49, 95% CI 1.48–4.18, *p* < 0.001).

Positive perceptions of benefits were also significant. Surgeons who believed RAS improves patient recovery time were nearly three times more likely to be supporters (aOR 2.93, 95% CI 1.14–7.49, *p* = 0.025). Interestingly, perceiving the cost of RAS to be higher than laparoscopic surgery was also a positive predictor of support (aOR 2.28, 95% CI 1.14–4.60, *p* = 0.021). Regarding demographics, younger age (aOR 0.96 per year, 95% CI 0.93–0.99, *p* = 0.013) and male gender (aOR 2.25, 95% CI 1.16–4.36, *p* = 0.016) were independent predictors of support.

### RAS patient cohort: demographics, perceptions, and procedural outcomes

The cohort of patients with direct RAS experience (*n* = 32) was distinct from the general patient pool: they were older (median age 45.5 years, IQR 23.8), had a higher proportion of females (65.6%), and exhibited higher levels of education (62.5% held a bachelor’s degree) and digital literacy. Full demographics are presented in Table 1.

### Knowledge and perceptions of RAS patients

Their source of knowledge was overwhelmingly their doctor (90.6%), resulting in a markedly more accurate understanding: 65.6% correctly identified the surgeon’s role, and 84.4% accurately recognized its similarity to laparoscopic surgery. Perceptions of RAS providers were overwhelmingly positive, with 96.9% believing RAS-trained surgeons are more skilled and 93.8% believing hospitals offering RAS are better.

### Experience and outcomes following RAS

The most common procedures were robotic-assisted hysterectomy (40.6%) and prostatectomy (31.3%), and the primary reason for choosing RAS was surgeon recommendation (46.9%). Patient-reported outcomes were highly positive (Table 5). The vast majority rated their overall experience as “Excellent” (62.5%) or “Very Good” (21.9%). Recovery was perceived favorably; 50.0% reported a faster recovery and 46.9% returned to regular activities within a month. Satisfaction with cosmetic outcomes was high (65.6% “Excellent”). All patients found RAS at least “Effective,” with 68.8% rating it “Highly Effective.” The majority (62.5%) reported no complications. This positive experience translated into strong advocacy; 90.6% of patients stated they would advise others to undergo RAS.

**Table 5:** RAS patients’ knowledge, perceptions, and experience

	Overall (*N* = 32)
**Source of knowledge about RAS (Select all that apply)**	
Internet and social media	3 (9.4%)
Doctor	29 (90.6%)
Friends and relatives	1 (3.1%)
Hospital	4 (12.5%)
**Understanding of robotic surgery**	
Robot performs surgery, trained surgeon stands by	5 (15.6%)
Surgeon controls robotic arms and instrument	21 (65.6%)
Surgeon tells robot what to do, robot follows each command	5 (15.6%)
I don’t know	1 (3.1%)
**Which type of surgery is robotic surgery most similar to?**	
Laparoscopic surgery	27 (84.4%)
Laser surgery	2 (6.2%)
I don’t know	3 (9.4%)
**Perceptions of surgeons trained in robotic surgery**	
Less skilled	1 (3.1%)
More skilled	31 (96.9%)
**Perceptions of hospitals using robotic surgery**	
Similar	2 (6.2%)
Better	30 (93.8%)
**Robotic surgery can replace the currently used surgical procedures**	
Yes	17 (53.1%)
No	3 (9.4%)
Not sure	12 (37.5%)
**The use of robotics in surgeries can improve surgical outcomes**	
Yes	28 (87.5%)
Not sure	4 (12.5%)
**Type of RAS performed**	
Robotic-assisted excision of endometriosis	4 (12.5%)
Robotic-assisted excision of endometriosis and fibroid	1 (3.1%)
Robotic-assisted excision of endometriosis and ovarian cysts	1 (3.1%)
Robotic-assisted excision of endometriosis, ovarian cysts, and endometrial ablation	1 (3.1%)
Robotic-assisted excision of ovarian cysts	2 (6.2%)
Robotic-assisted hysterectomy	13 (40.6%)
Robotic-assisted prostatectomy	10 (31.3%)
**The main cause of choosing RAS**	
Safer	13 (40.6%)
Less pain	2 (6.3%)
Recovery faster	1 (3.1%)
More precise	1 (3.1%)
The surgeon recommended it	15 (46.9%)
**In general**,** how was your experience with RAS**	
Bad	1 (3.1%)
Good	4 (12.5%)
Very good	7 (21.9%)
Excellent	20 (62.5%)
**Level of pain within the first week after RAS**	
No pain	3 (9.4%)
Mild pain	11 (34.4%)
Moderate pain	13 (40.6%)
Severe pain	5 (15.6%)
**Time of recovery from RAS compared to other surgeries**	
Much slower	1 (3.1%)
About the same duration	4 (12.5%)
Faster	16 (50%)
Much faster	11 (34.4%)
**Time to return to regular activities after RAS**	
Within a few days	4 (12.5%)
Within a week	10 (31.2%)
Within a month	15 (46.9%)
More than a month	3 (9.4%)
**Personal assessment of the size of the wound and scars after RAS**	
Very bad	1 (3.1%)
Bad	1 (3.1%)
Good	2 (6.2%)
Very good	7 (21.9%)
Excellent	21 (65.6%)
**Effectiveness in terms of solving the medical problem**	
Not effective	0 (0)
Effective	10 (31.2%)
Highly effective	22 (68.8%)
**Level of complications after RAS**	
No complications	20 (62.5%)
Mild complications	6 (18.8%)
Moderate complications	5 (15.6%)
Severe complications	1 (3.1%)
**Do you advise others to perform RAS surgery if they need it**	
Yes	29 (90.6%)
Not sure	3 (9.4%)

## Discussion

This national, multi-cohort study provides the first comprehensive analysis of the landscape for robotic-assisted surgery (RAS) in Egypt, revealing that the path to adoption is dominated not by demographics but by a critical and modifiable perception gap. Our findings indicate a gradient: surgeons are broadly supportive yet constrained by knowledge gaps, general patients are hesitant due to fear and misinformation, and crucially, RAS-experienced patients become powerful advocates after direct exposure. This pattern suggests that strategic education and exposure could help narrow this divide and may provide a useful framework for other LMICs.

### The perception gap as a primary barrier to patient acceptance

The strikingly low awareness of RAS among Egyptian patients (30.4%) and their profound fears of malfunction and error present a formidable barrier to adoption. This finding is consistent with regional trends; in Kuwait, public awareness was reported at 37% with similar uncertainties [14], and in Saudi Arabia, despite higher awareness (51%), fears of malfunction and misperceptions about safety were prevalent [19]. Our study significantly advances this understanding by quantifying the devastating impact of these fears through multivariable analysis, identifying them as the single strongest predictor of patient refusal—those with fears were less likely to prefer RAS (aOR 0.08). This provides robust, empirical evidence that perceptual barriers can be more immediately limiting than financial ones in shaping patient demand. The fact that patients primarily learned about RAS from the internet and social media (78.0%) underscores a critical vulnerability: a vacuum of formal, reliable patient education that is being filled by potentially misleading sources, a challenge also noted in other settings [9]. This reliance on unvetted information likely exacerbates misconceptions and underscores the urgent need for structured educational initiatives led by healthcare institutions.

### The surgeon’s dilemma: enthusiasm hampered by systemic constraints

In contrast to patients, surgeon awareness was nearly universal (90.7%). However, this awareness often lacked depth, revealing a significant “know-do” gap. Critical knowledge gaps—such that only 56.2% understood the surgeon maintains complete control—mirror misconceptions found among surgeons in Kuwait, where similar misunderstandings about autonomy were common [12]. The finding that a substantial minority of surgeons did not indicate full surgeon control over robotic arms likely reflects limited hands-on exposure and conflation of ‘robotic-assisted’ platforms with autonomous automated systems in the broader discourse on artificial intelligence (AI) and automation. Surgeons without direct experience may infer higher degrees of autonomy from media narratives around AI. This highlights the need for structured technical education that emphasizes system architecture, intraoperative surgeon control, and the limitations of current automation.

Generational differences in surgical training and exposure appear to underlie part of the observed variation in attitudes toward robotic-assisted surgery. Younger surgeons, particularly residents and early-career specialists, were more supportive of adopting RAS and demonstrated relatively higher awareness of its capabilities. This likely reflects evolving surgical curricula that now include simulation-based training, laparoscopic proficiency, and exposure to emerging robotic systems, even if only observationally. In contrast, senior surgeons may have completed their core training before the widespread integration of minimally invasive or digital technologies, which could explain their lower familiarity and more cautious stance. Bridging this generational training gap through structured workshops, short observerships, and hands-on simulation may therefore be an important consideration for accelerating nationwide RAS adoption.

Our analysis reveals a more profound systemic barrier: the strongest predictor of a surgeon supporting national implementation was simply the knowledge that RAS is already available in Egypt (aOR 6.35). This reveals a crucial insight: a major impediment is not just the objective high cost [8], but the widespread perceived inaccessibility and lack of visibility. Surgeons in hospitals without RAS were significantly less informed and less supportive, highlighting how uneven resource distribution creates a self-perpetuating cycle where limited access begets limited knowledge and lukewarm advocacy. This phenomenon, where potential champions are neutralized by a lack of exposure, has been observed in other emerging RAS markets like the UAE [20]. Therefore, the surgeon cohort represents not a barrier, but an untapped reservoir of potential advocates who require integrated training programs and first-hand exposure to become effective change agents.

### The power of direct exposure: a blueprint for change

The experience of the RAS-patient cohort offers important insight into how perception gaps might be addressed. Their transition from limited awareness to strong advocacy provides a useful illustration of the influence of direct exposure. Unlike the general patients, most patients in this group (90.6%) reported that their primary source of information was their surgeon, which appeared to foster greater trust and a more accurate understanding of the technology before surgery. This finding highlights the critical role of clinicians as trusted communicators in shaping perceptions of new surgical innovations. After surgery, patients reported very high satisfaction (84.4% rating their experience as excellent or very good), perceived faster recovery, and few complications—patterns consistent with international findings of high satisfaction following RAS [21, 22]. These results suggest that positive, first-hand experience and surgeon-led education may help reduce fear and misperceptions among prospective patients. Although causal relationships cannot be inferred from this cross-sectional design, the contrast between RAS-experienced and general patient groups indicates that structured education and exposure could be promising strategies to improve acceptance.

### Beyond Egypt: implications for surgical innovation in LMICs

The consistency of our findings with reports from other MENA countries indicates that the “perception gap” may represent a common, yet often under-recognized, phase in the diffusion of surgical innovation in LMICs [13, 17]. The approach used in this study, simultaneously assessing the awareness and attitudes of key stakeholders along the adoption pathway, could serve as a useful framework for other countries seeking to evaluate their readiness and barriers. The prominent influence of surgeon recommendation on patient decision-making, observed both here and elsewhere [9], underscores that technological adoption is not purely technical but also social, relying heavily on trust between clinicians and patients. Consequently, national implementation strategies would ideally address both dimensions: strengthening technical capacity while concurrently shaping public and professional perceptions through transparent communication and education.

### Implications for policy and practice

The present findings suggest several potential directions for a national strategy to enhance awareness and acceptance of robotic-assisted surgery. First, developing national educational campaigns could help address and clarify common fears, such as concerns about malfunction or loss of surgeon control, while sharing positive outcomes and experiences from local patients. Second, strengthening surgeon training and exposure through structured, hands-on programs and partnerships between hospitals with and without RAS systems may build a wider base of informed advocates and help reduce existing knowledge gaps. Third, incorporating patient testimonials in educational materials, waiting areas, and clinical consultations could provide relatable peer perspectives that help ease pre-operative anxiety. Finally, strategic institutional communication, including transparent promotion of outcomes and services by hospitals offering RAS, may improve public and professional awareness of system availability and capability.

### Strengths and limitations

This study has several strengths. It represents the first national, multi-center analysis of perceptions toward robotic-assisted surgery (RAS) in Egypt, incorporating insights from both key stakeholder groups, surgeons and patients, and including a unique cohort of individuals with direct RAS experience. The use of validated, pilot-tested questionnaires and multivariable regression analysis strengthens methodological rigor and enhances internal validity.

Several limitations, however, should be acknowledged. First, the use of non-probability (convenience) sampling introduces potential selection bias and limits the representativeness of the study population, particularly for the general patient and surgeon cohorts. Second, the relatively small sample of RAS-experienced patients restricts the precision and generalizability of their responses, and their highly positive feedback may partially reflect response or recall bias. Third, as participation was voluntary and data were self-reported, both social desirability and information bias cannot be excluded, especially among surgeons who may have felt compelled to express favorable views. Fourth, response rates for general patients and surgeons could not be precisely calculated, limiting the assessment of potential non-response bias. Fifth, the cross-sectional design captures perceptions at a single point in time and therefore cannot establish causality or temporal changes in awareness and attitudes. Finally, because this study focused on governmental university hospitals, findings may not fully reflect the views of private-sector surgeons or patients in rural and non-academic settings.

Despite these limitations, the consistency of observed trends with reports from other MENA countries strengthens confidence in the external relevance of the findings and highlights the need for future longitudinal and interventional studies as RAS adoption expands.

## Conclusion

In conclusion, the integration of robotic-assisted surgery (RAS) in Egypt and comparable LMICs appears to depend not only on financial investment but also on addressing the broader perceptual and educational challenges that shape acceptance. This study identifies a measurable perception gap among both surgeons and patients and highlights education and exposure as potential strategies to reduce it. Targeted, trust-based initiatives aimed at improving understanding and first-hand familiarity may help foster greater support and informed decision-making. Future longitudinal and interventional research is needed to confirm whether such strategies translate into sustained improvements in acceptance and equitable access to RAS.

## Supplementary Information

Below is the link to the electronic supplementary material.

Supplementary Material 1

Supplementary Material 2

Supplementary Material 3

Supplementary Material 4

## Data Availability

The data used in this study are available.

## Abbreviations

RAS: Robotic-assisted surgery
LMIC: Low- and middle-income countries
MENA: Middle East and North Africa
RECs: Research Ethics Committees
RAS patients: Patients underwent robotic-assisted surgery
SD: Standard deviation
IQR: Interquartile range
aOR: Adjusted Odds Ratio

## References

[1] Kirkpatrick T, LaGrange C (2017) Robotic surgery: risks vs. Rewards. AORN J 106(2):186. 10.1016/j.aorn.2017.05.007 28755672 10.1016/j.aorn.2017.05.007

[2] Panait L, Doarn CR, Merrell RC (2002) Applications of robotics in surgery. Chirurgia (Bucur). ;97(6):549 – 55. PMID: 12731212. Available from: https://pubmed.ncbi.nlm.nih.gov/12731212/12731212

[3] Hussain A, Malik A, Halim MU, Ali AM (2014) The use of robotics in surgery: a review. Int J Clin Pract 68(11):1376–82. 10.1111/ijcp.12492 25283250 10.1111/ijcp.12492

[4] Van Koughnett JA, Jayaraman S, Eagleson R et al (2009) Are there advantages to robotic-assisted surgery over laparoscopy from the surgeon’s perspective? J Robotic Surg 3:79–82. 10.1007/s11701-009-0144-8 10.1007/s11701-009-0144-8 27638219

[5] Intuitive Surgical JP, Morgan Healthcare C (2023) [(accessed on 23 May 2023)]. Available online: https://isrg.intuitive.com/static-files/6683d2bb-75e2-4fa0-b0cd-463ead7c30a4

[6] Sheetz KH, Claflin J, Dimick JB (2020) Trends in the adoption of robotic surgery for common surgical procedures. JAMA Netw Open 3(1):e1918911. 10.1001/jamanetworkopen.2019.18911 31922557 10.1001/jamanetworkopen.2019.18911 PMC6991252

[7] Burke J, Gnanaraj J, Dhanda J, Martins B, Vinck EE, Saklani A, Harji D (2024) Robotic surgery in low- and middle-income countries. Bull R Coll Surg Engl 106(3):138–141. 10.1308/rcsbull.2024.54

[8] Mehta A, Ng JC, Awuah WA, Huang H, Kalmanovich J, Agrawal A, Abdul-Rahman T, Hasan MM, Sikora V, Isik A (2022) Embracing robotic surgery in low- and middle-income countries: potential benefits, challenges, and scope in the future. Annals Med Surg. 10.1016/j.amsu.2022.104803 10.1016/j.amsu.2022.104803 PMC9793116 36582867

[9] Jauniaux B, Anand A, Abbas R, Harji DP (2025) From expectations to experiences: a systematic review of patient and public perspectives on robotic surgery. J Robot Surg 19:484. 10.1007/s11701-025-02649-y 40810856 10.1007/s11701-025-02649-y PMC12354569

[10] BenMessaoud C, Kharrazi H, MacDorman KF (2011) Facilitators and barriers to adopting robotic-assisted surgery: contextualizing the unified theory of acceptance and use of technology. PLoS ONE 6(1):e16395. 10.1371/journal.pone.0016395 21283719 10.1371/journal.pone.0016395 PMC3024425

[11] Brar G, Xu S, Anwar M, Talajia K, Ramesh N, Arshad SR (2024) Robotic surgery: public perceptions and current misconceptions. J Robot Surg 18(1):84. 10.1007/s11701-024-01837-6 38386115 10.1007/s11701-024-01837-6 PMC10884196

[12] Aldousari SA, Buabbas AJ, Yaiesh SM, Alyousef RJ, Alenezi AN (2021) Multiple perceptions of robotic-assisted surgery among surgeons and patients: a cross-sectional study. J Robot Surg 15(4):529–538. 10.1007/s11701-020-01136-w 32776285 10.1007/s11701-020-01136-w

[13] Al Dihan FA, Alghamdi MA, Aldihan FA, Alamer NM, Alshahrani FA, Alqarni A (2024) Knowledge, Attitude, Awareness, and Future Expectations of Robotic Surgery in Patients Attending Surgical Specialties Clinics. Cureus. 10.7759/cureus.56523 10.7759/cureus.56523 PMC11027023 38646294

[14] Buabbas AJ, Aldousari S, Shehab AA (2020) An exploratory study of public’ awareness about robotics-assisted surgery in Kuwait. BMC Med Inform Decis Mak 20:140. 10.1186/s12911-020-01167-1 32611407 10.1186/s12911-020-01167-1 PMC7329483

[15] Egypt’s Qasr El- Aini Hospital begins performing robotic surgeries-SIS https://www.sis.gov.eg/Story/170951/Egypt’s-Qasr-El-Aini-Hospital-begins-performing-robotic-surgeries?lang=en-us

[16] Abd-erRazik MA, Abdel Hamid MA, Rashed AM (2022) Egypt’s Initial Experience With Robotic-Assisted Cystogastrostomy and Pancreatic Debridement for Large Walled-Off Pancreatic Necrosis: A Report of Two Cases. Cureus. 10.7759/cureus.32005 10.7759/cureus.32005 PMC9798344 36589168

[17] Boys JA, Alicuben ET, DeMeester MJ, Worrell SG, Oh DS, Hagen JA, DeMeester SR (2016) Public perceptions on robotic surgery, hospitals with robots, and surgeons that use them. Surg Endosc 30(4):1310–1316. 10.1007/s00464-015-4368-6 26173543 10.1007/s00464-015-4368-6

[18] Charan J, Biswas T (2013) How to calculate sample size for different study designs in medical research? Indian J Psychol Med 35(2):121–126. 10.4103/0253-7176.116232 24049221 10.4103/0253-7176.116232 PMC3775042

[19] Arishi AA, Hakami IA, Mashbari HN, Hobani AH, Al-Musawa HI, Abuhadi RI, Maslouf AH, Matari MH, Albrahim HT, Algarni MA, Iskander O, Alyahyawi K (2024) Knowledge, attitude, and perception of robotic-assisted surgery among the general population in Saudi Arabia: a cross-sectional study. J Robot Surg 18:196. 10.1007/s11701-024-01892-z 38703278 10.1007/s11701-024-01892-z

[20] Barkati N, Ntefeh N, Okasha A, Takshe AA, ElKhatib R, Chelli S (2023) Robotic assisted surgery in the United Arab Emirates: healthcare experts’ perceptions. J Robot Surg 17(6):2799–2806. 10.1007/s11701-023-01716-6 37733210 10.1007/s11701-023-01716-6 PMC10678779

[21] Muniasamy K, Sivakumar A, Rameshbabu KN, Ganesan A, Rajkumar VA, Deshmukh GV, Sanjana SV, Sharma S (2024) Patient satisfaction and quality of life outcomes following robotic-assisted surgery: a survey-based study. Bioinformation 20(12):1964–1969. 10.6026/9732063002001964 40230909 10.6026/9732063002001964 PMC11993418

[22] Park S, Lee Y-E, Cho S-S, Park S-H, Park S-T (2018) Patient-reported satisfaction after robot-assisted hysterectomy among Korean patients with benign uterine disease. Obstet Gynecol Sci 61(6):675–683. 10.5468/ogs.2018.61.6.675 30474014 10.5468/ogs.2018.61.6.675 PMC6236087

