# Supplementary material for: Robotic-assisted surgery in Egypt: national insights into awareness, knowledge, and perceptions among surgeons and patients
Source: J Robot Surg. 2025 Nov 21;20(1):32. doi: 10.1007/s11701-025-02942-w (PMC12634703; doi:10.1007/s11701-025-02942-w)
Supplement: Supplementary file 1 — Supplementary Material 1 [file 11701_2025_2942_MOESM1_ESM.docx]

**Journal of Robotic Surgery**

**“Questionnaire assessing surgeons’ knowledge and perceptions of robotic-assisted surgery.”**

**Robotic-Assisted Surgery in Egypt: National Insights into Awareness, Knowledge, and Perceptions Among Surgeons and Patients**

Mohamed F. Srour^1^, Ahmed H. Shoaib^2^, Hazim Alkousheh^3^, Karim K. Eladawy^4^, Mohamed Alayat^5^, Ahmed Abdelhameed^5^, Osama Alhaddad^4^, Seif M. Elsadik^6^, Ezzeldin Ahmed Abdelaty^7^, Mohamed Sloma^5^, Nada Rady^6^, Mohammad A. Abd-erRazik^8^

*Affiliations*

1. Faculty of Medicine, Menoufia University, Menoufia, Egypt.

ORCID: 0009-0008-4305-1971

1. Faculty of Medicine, Alexandria University, Alexandria, Egypt.

ORCID: 0009-0005-0708-6569

1. Faculty of Medicine, The Hashemite University, Zarqa, Jordan.

ORCID: 0009-0003-7175-7529

1. Faculty of Medicine, Ain Shams University, Cairo, Egypt.

ORCID (K.K.E): 0009-0002-9032-7017

ORCID (O.A): 0009-0003-7454-1838

1. Faculty of Medicine, Al Azhar University, Cairo, Egypt.

ORCID (M.A): 0009-0008-0183-6869

ORCID (A.A): 0009-0006-5491-1143

ORCID (M.S): 0009-0001-8998-918

1. Faculty of Medicine, 6th of October University, Giza, Egypt

ORCID (S.M.E): 0009-0007-1362-9923

ORCID (N.R): 0009-0003-5788-9082

1. Faculty of Medicine, Al-Azhar University, Damietta, Egypt.

ORCID: 0009-0004-4552-6919

1. General Surgery, Faculty of Medicine, Ain Shams University, Cairo, Egypt.

ORCID: 0000-0002-8498-9957

*Correspondence*

Mohamed F. Srour, MBBCh

Faculty of Medicine, Menoufia University

Address: Menoufia - Egypt

Phone Number: +201279012239

E-mail: [mohamed.ft.srour@gmail.com](mailto:mohamed.ft.srour@gmail.com)

**Revised Questionnaire for Surgeons**

**Instructions:** Please tick (✓) the most appropriate answer for each question.

*Note: RAS refers to robotic-assisted surgery.*

**Section A: Demographic Information and Experience with Technology**

**Name:** ........................................

1. **What is your age?**
   - ………………… years
2. **What is your gender?**
   - □ Male
   - □ Female
3. **What is your nationality?**
   - □ Egyptian
   - □ Non-Egyptian
4. **What is your current specialty?**
   - □ General Surgery
   - □ Urology
   - □ Obstetrics and Gynaecology
   - □ Cardiothoracic Surgery
   - □ Orthopedic Surgery
   - □ ENT
   - □ Neurosurgery
   - □ Pediatric Surgery
   - □ Vascular Surgery
   - □ Other (please specify): ........................................
5. **What is your current employment level?**
   - □ Consultant
   - □ Specialist / Assistant Lecturer
   - □ Registrar/Senior Resident
   - □ Assistant Registrar/Junior Resident
   - □ Trainee
6. **How many hours do you use a computer daily?**

Approximately ………… hours/day

1. **What is your level of computer proficiency?**

- □ None: Cannot use a computer
- □ Beginner: Can perform basic tasks like starting the computer, browsing the internet, and using email
- □ Intermediate: Can use productivity software (e.g., Word, Excel) and solve basic technical issues
- □ Advanced: Can manage complex software, troubleshoot systems, and use advanced features of applications
- □ Expert: Can write or customize software programs and manage IT infrastructure

**Section B: Surgeons’ Knowledge and Perceptions About Robotic-Assisted Surgery (RAS)**

1. **Have you heard of robotic-assisted surgery (RAS)?**
   - □ Yes
   - □ No (If "No," skip to Question 10)
2. **Where did you hear about robotic-assisted surgery?** (Select all that apply):
   - □ During undergraduate studies
   - □ Medical conferences
   - □ Colleagues
   - □ Residency or fellowship training programs
   - □ Internet and social media
   - □ Medical journals
   - □ Other (please specify): ........................................
3. **Which statement best aligns with your understanding of robotic-assisted surgery?**
   - □ The robot performs the surgery while a trained surgeon supervises.
   - □ The surgeon controls the robotic arms and performs all surgical tasks.
   - □ The surgeon programs the robot to perform specific moves.
   - □ The surgeon issues commands, and the robot executes them.
4. **What is the level of surgeon control during RAS?**
   - □ Complete control
   - □ Major control
   - □ Minimal control
   - □ No control
   - □ Not sure
5. **Do you support introducing RAS into the healthcare system?**
   - □ Agree
   - □ Disagree
   - □ Not sure (skip to Question 14)
6. **Reasons for your opinion:**
   - **Reasons to agree with RAS adoption:**
     1. □ More precise performance intraoperatively
     2. □ Improved magnification and 3D vision
     3. □ Reduced complications
     4. □ Better outcomes
     5. □ Faster than open and laparoscopic surgery
     6. □ Attracts more patients
     7. □ Fashionable
     8. □ Other (please specify): ........................................
   - **Reasons to disagree with RAS adoption:**
     1. □ Laparoscopic surgery achieves similar outcomes
     2. □ Limited availability
     3. □ High cost
     4. □ Difficult to learn
     5. □ Increased complication rates
     6. □ Other (please specify): ........................................
7. **Is robotic-assisted surgery currently available in Egypt?**
   - □ Yes
   - □ No
   - □ I don’t know

**Section C: Surgeons’ Knowledge and Attitudes of RAS^*^ compared to other surgical approaches**

| **15. Time spent discussing RAS as an alternative option to:** | **Much less** | **Less** | **Same** | **More** | **Much more** | **NA** |
| --- | --- | --- | --- | --- | --- | --- |
| Open surgery |  |  |  |  |  |  |
| Laparoscopic surgery |  |  |  |  |  |  |

| **16. Frequency of recommending RAS as an alternative option to:** | **Never** | **Infrequently** | **Frequently** | **Very frequently** | **Every time** | **NA** |
| --- | --- | --- | --- | --- | --- | --- |
| Open surgery |  |  |  |  |  |  |
| Laparoscopic surgery |  |  |  |  |  |  |

| **17. Learning curve of RAS compared to:** | **Dramatically slower** | **Slower** | **Similar** | **Faster** | **Dramatically faster** | **Not sure** |
| --- | --- | --- | --- | --- | --- | --- |
| Open surgery |  |  |  |  |  |  |
| Laparoscopic surgery |  |  |  |  |  |  |

| **18. The Overall cost of RAS compared to laparoscopic surgery:** | **Dramatically less** | **Less** | **Similar** | **More** | **Dramatically more** | **Not sure** |
| --- | --- | --- | --- | --- | --- | --- |
|  |  |  |  |  |  |  |

| **19. The effect of RAS on:** | **Dramatically decreases** | **Decreases** | **Remains the same** | **Increases** | **Dramatically increases** | **Not sure** |
| --- | --- | --- | --- | --- | --- | --- |
| Operative time |  |  |  |  |  |  |
| Recovery time |  |  |  |  |  |  |

RAS robotic-assisted surgery, NA not applicable
