# Supplementary material for: Robotic-assisted surgery in Egypt: national insights into awareness, knowledge, and perceptions among surgeons and patients
Source: J Robot Surg. 2025 Nov 21;20(1):32. doi: 10.1007/s11701-025-02942-w (PMC12634703; doi:10.1007/s11701-025-02942-w)
Supplement: Supplementary file 2 — Supplementary Material 2 [file 11701_2025_2942_MOESM2_ESM.docx]

**Journal of Robotic Surgery**

**“Questionnaire assessing patients’ awareness and perceptions of robotic-assisted surgery.”**

**Robotic-Assisted Surgery in Egypt: National Insights into Awareness, Knowledge, and Perceptions Among Surgeons and Patients**

Mohamed F. Srour^1^, Ahmed H. Shoaib^2^, Hazim Alkousheh^3^, Karim K. Eladawy^4^, Mohamed Alayat^5^, Ahmed Abdelhameed^5^, Osama Alhaddad^4^, Seif M. Elsadik^6^, Ezzeldin Ahmed Abdelaty^7^, Mohamed Sloma^5^, Nada Rady^6^, Mohammad A. Abd-erRazik^8^

*Affiliations*

1. Faculty of Medicine, Menoufia University, Menoufia, Egypt.

ORCID: 0009-0008-4305-1971

1. Faculty of Medicine, Alexandria University, Alexandria, Egypt.

ORCID: 0009-0005-0708-6569

1. Faculty of Medicine, The Hashemite University, Zarqa, Jordan.

ORCID: 0009-0003-7175-7529

1. Faculty of Medicine, Ain Shams University, Cairo, Egypt.

ORCID (K.K.E): 0009-0002-9032-7017

ORCID (O.A): 0009-0003-7454-1838

1. Faculty of Medicine, Al Azhar University, Cairo, Egypt.

ORCID (M.A): 0009-0008-0183-6869

ORCID (A.A): 0009-0006-5491-1143

ORCID (M.S): 0009-0001-8998-918

1. Faculty of Medicine, 6th of October University, Giza, Egypt

ORCID (S.M.E): 0009-0007-1362-9923

ORCID (N.R): 0009-0003-5788-9082

1. Faculty of Medicine, Al-Azhar University, Damietta, Egypt.

ORCID: 0009-0004-4552-6919

1. General Surgery, Faculty of Medicine, Ain Shams University, Cairo, Egypt.

ORCID: 0000-0002-8498-9957

*Correspondence*

Mohamed F. Srour, MBBCh

Faculty of Medicine, Menoufia University

Address: Menoufia - Egypt

Phone Number: +201279012239

E-mail: [mohamed.ft.srour@gmail.com](mailto:mohamed.ft.srour@gmail.com)

**القسم 1: المعلومات الشخصية والخبرة بالتكنولوجيا**

1. الاسم .....................................................
2. العمر .................................................... عامًا
3. الجنس:
   - □ ذكر
   - □ أنثى
4. الجنسية:
   - □ مصري
   - □ غير مصري (يرجى التحديد ....................(
5. المستوى التعليمي:
   - □ ابتدائي
   - □ ثانوي
   - □ دبلوم
   - □ جامعي
   - □ دراسات عليا
6. كم ساعة في المتوسط تستخدم التكنولوجيا يوميًا (مثل أجهزة الكمبيوتر، الهواتف المحمولة، إلخ)؟
   - ............................. ساعة/اليوم
7. ما مدى راحتك في التعامل مع التكنولوجيا؟
   - □ غير مريح
   - □ مريح إلى حد ما
   - □ مريح
8. كيف تصنف خبرتك في استخدام التكنولوجيا؟
   - □ مبتدئ (ليس لدي خبرة)
   - □ متوسط (لدي خبرة)
   - □ متقدم (محترف)

**القسم 2: المعرفة بالجراحة الروبوتية**

1. هل سمعت من قبل عن الجراحة الروبوتية؟
   - □ نعم
   - □ لا (انتقل إلى السؤال رقم 11)
2. إذا كانت الإجابة "نعم"، من أين سمعت عنها؟ (اختر كل ما ينطبق)
   - □ الإنترنت ومواقع التواصل الاجتماعي
   - □ الطبيب
   - □ وسائل الإعلام (تلفزيون، إذاعة، إلخ)
   - □ مجلة
   - □ الأقارب أو الأصدقاء
   - □ غير ذلك (يرجى التحديد): ....................
3. ما الذي تفهمه من مصطلح "الجراحة الروبوتية"؟
   - □ الروبوت يجري الجراحة بينما الجراح يراقب
   - □ الجراح يتحكم في الأدوات الروبوتية ويجرى العملية بالكامل
   - □ الروبوت يتبع أوامر مبرمجة مسبقًا من الجراح
   - □ الجراح غير موجود في غرفة العمليات والروبوت يجري العملية وفق البرمجة
   - □ لا أعرف
4. أي من الخيارات التالية تتشابه أكثر مع الجراحة الروبوتية؟
   - □ الجراحة التقليدية المفتوحة (قطع كبير في الجلد)
   - □ جراحة المناظير (جراحة بفتحات صغيرة)
   - □ جراحة الليزر
   - □ لا أعرف
5. هل الجراحة الروبوتية متوفرة في مصر؟
   - □ نعم
   - □ لا
   - □ لست متأكدًا

**القسم 3: التقييم الشخصي للجراحة الروبوتية**

1. مقارنة بالجراحة التقليدية، ما الذي يرتبط باستخدام الجراحة الروبوتية؟ (اختر كل ما ينطبق):
   - □ أكثر أمانًا
   - □ أقل ألمًا
   - □ نتائج أفضل
   - □ أسرع
   - □ أكثر تكلفة
   - □ لا شيء مما سبق
2. هل لديك أي مخاوف بشأن الجراحة الروبوتية؟ (اختر كل ما ينطبق):
   - □ تعطل الروبوت أثناء الجراحة
   - □ الروبوت قد يقوم بعملية خاطئة
   - □ ليس لدي مخاوف
   - □ غير ذلك (يرجى التحديد): ....................
3. إذا كنت بحاجة إلى عملية جراحية، هل تفضل استخدام الروبوت؟
   - □ نعم
   - □ لا
   - □ لست متأكدًا
4. ما رأيك في مهارة الجراحين الذين يستخدمون الروبوت مقارنة بغيرهم؟
   - □ أكثر مهارة
   - □ متشابهة في المهارة
   - □ أقل مهارة
5. ما رأيك في المستشفيات التي تستخدم الروبوت؟
   - □ أفضل من المستشفيات التي لا تستخدم الروبوت
   - □ مشابهة للمستشفيات التي لا تستخدم الروبوت
   - □ أسوأ من المستشفيات التي لا تستخدم الروبوت
6. مقارنة بجراحة المناظير القياسية، هل الجراحة الروبوتية:
   - □ أكثر تكلفة
   - □ نفس التكلفة
   - □ أقل تكلفة
7. هل تعتقد أن الجراحة الروبوتية ستحل محل العمليات الجراحية التقليدية؟
   - □ نعم
   - □ لا
   - □ لست متأكدًا
8. هل تعتقد أن استخدام الروبوت في العمليات يمكن أن يحسن النتائج الجراحية؟
   - □ نعم
   - □ لا
   - □ لست متأكدًا

**English version of general patient questionnaire**

## **Section 1: Demographics and Technology Experience**

1. **Name**
   _______________________________________________________
2. **Age**
   _______________________________________________________ years
3. **Gender**:
   - ⬜ Male
   - ⬜ Female
4. **Nationality**:
   - ⬜ Egyptian
   - ⬜ Non-Egyptian (please specify) ____________________________
5. **Education Level**:
   - ⬜ Primary
   - ⬜ Secondary
   - ⬜ Diploma
   - ⬜ University
   - ⬜ Postgraduate
6. **On average, how many hours per day do you use technology (e.g., computers, mobile phones, etc.)?**
   ____________________________ hours/day
7. **How comfortable are you with using technology?**
   - ⬜ Not comfortable
   - ⬜ Somewhat comfortable
   - ⬜ Comfortable
8. **How would you classify your experience with technology?**
   - ⬜ Beginner (no experience)
   - ⬜ Intermediate (some experience)
   - ⬜ Advanced (professional)

## **Section 2: Knowledge of Robotic Surgery**

1. **Have you ever heard of robotic surgery?**
   - ⬜ Yes
   - ⬜ No (skip to Question 11)
2. **If "Yes," where did you hear about it? (Select all that apply)**
   - ⬜ Internet/Social Media
   - ⬜ Doctor
   - ⬜ Media (TV, radio, etc.)
   - ⬜ Magazine
   - ⬜ Relatives/Friends
   - ⬜ Other (please specify) ____________________________
3. **What do you understand by the term "robotic surgery"?**
   - ⬜ The robot performs the surgery while the surgeon observes
   - ⬜ The surgeon controls robotic tools and performs the entire procedure
   - ⬜ The robot follows pre-programmed commands from the surgeon
   - ⬜ The surgeon is not present in the operating room, and the robot performs the surgery autonomously
   - ⬜ I don’t know
4. **Which of the following is most similar to robotic surgery?**
   - ⬜ Traditional open surgery (large incision)
   - ⬜ Laparoscopic surgery (minimally invasive with small incisions)
   - ⬜ Laser surgery
   - ⬜ I don’t know
5. **Is robotic surgery available in Egypt?**
   - ⬜ Yes
   - ⬜ No
   - ⬜ I’m not sure

## **Section 3: Perceptions of Robotic Surgery**

1. **Compared to traditional surgery, what do you associate with robotic surgery? (Select all that apply)**
   - ⬜ Safer
   - ⬜ Less painful
   - ⬜ Better outcomes
   - ⬜ Faster
   - ⬜ More expensive
   - ⬜ None of the above
2. **Do you have any concerns about robotic surgery? (Select all that apply)**
   - ⬜ Robot malfunction during surgery
   - ⬜ The robot may make a mistake
   - ⬜ I have no concerns
   - ⬜ Other (please specify) ____________________________
3. **If you needed surgery, would you prefer robotic surgery?**
   - ⬜ Yes
   - ⬜ No
   - ⬜ I’m not sure
4. **What do you think about the skill of surgeons who use robots compared to those who don’t?**
   - ⬜ More skilled
   - ⬜ Equally skilled
   - ⬜ Less skilled
5. **What is your opinion of hospitals that use robotic surgery?**
   - ⬜ Better than hospitals that don’t use robots
   - ⬜ Similar to hospitals that don’t use robots
   - ⬜ Worse than hospitals that don’t use robots
6. **Compared to standard laparoscopic surgery, robotic surgery is:**
   - ⬜ More expensive
   - ⬜ Same cost
   - ⬜ Less expensive
7. **Do you think robotic surgery will replace traditional surgery in the future?**
   - ⬜ Yes
   - ⬜ No
   - ⬜ I’m not sure
8. **Do you believe robotic surgery can improve surgical outcomes?**
   - ⬜ Yes
   - ⬜ No
   - ⬜ I’m not sure
