# Supplementary material for: Robotic-assisted surgery in Egypt: national insights into awareness, knowledge, and perceptions among surgeons and patients
Source: J Robot Surg. 2025 Nov 21;20(1):32. doi: 10.1007/s11701-025-02942-w (PMC12634703; doi:10.1007/s11701-025-02942-w)
Supplement: Supplementary file 3 — Supplementary Material 3 [file 11701_2025_2942_MOESM3_ESM.docx]

**Journal of Robotic Surgery**

**“Questionnaire assessing RAS patients’ perception and experience of robotic-assisted surgery.”**

**Robotic-Assisted Surgery in Egypt: National Insights into Awareness, Knowledge, and Perceptions Among Surgeons and Patients**

Mohamed F. Srour^1^, Ahmed H. Shoaib^2^, Hazim Alkousheh^3^, Karim K. Eladawy^4^, Mohamed Alayat^5^, Ahmed Abdelhameed^5^, Osama Alhaddad^4^, Seif M. Elsadik^6^, Ezzeldin Ahmed Abdelaty^7^, Mohamed Sloma^5^, Nada Rady^6^, Mohammad A. Abd-erRazik^8^

*Affiliations*

1. Faculty of Medicine, Menoufia University, Menoufia, Egypt.

ORCID: 0009-0008-4305-1971

1. Faculty of Medicine, Alexandria University, Alexandria, Egypt.

ORCID: 0009-0005-0708-6569

1. Faculty of Medicine, The Hashemite University, Zarqa, Jordan.

ORCID: 0009-0003-7175-7529

1. Faculty of Medicine, Ain Shams University, Cairo, Egypt.

ORCID (K.K.E): 0009-0002-9032-7017

ORCID (O.A): 0009-0003-7454-1838

1. Faculty of Medicine, Al Azhar University, Cairo, Egypt.

ORCID (M.A): 0009-0008-0183-6869

ORCID (A.A): 0009-0006-5491-1143

ORCID (M.S): 0009-0001-8998-918

1. Faculty of Medicine, 6th of October University, Giza, Egypt

ORCID (S.M.E): 0009-0007-1362-9923

ORCID (N.R): 0009-0003-5788-9082

1. Faculty of Medicine, Al-Azhar University, Damietta, Egypt.

ORCID: 0009-0004-4552-6919

1. General Surgery, Faculty of Medicine, Ain Shams University, Cairo, Egypt.

ORCID: 0000-0002-8498-9957

*Correspondence*

Mohamed F. Srour, MBBCh

Faculty of Medicine, Menoufia University

Address: Menoufia - Egypt

Phone Number: +201279012239

E-mail: [mohamed.ft.srour@gmail.com](mailto:mohamed.ft.srour@gmail.com)

**القسم 1: المعلومات الشخصية والخبرة بالتكنولوجيا**

1. الاسم .....................................................:
2. العمر .....................................................: عامًا
3. الجنس:
   - □ ذكر
   - □ أنثى
4. الجنسية:
   - □ مصري
   - □ غير مصري
5. المستوى التعليمي:
   - □ ابتدائي
   - □ إعدادي
   - □ ثانوي
   - □ دبلوم
   - □ جامعي
   - □ دراسات عليا
6. كم ساعة في المتوسط تستخدم التكنولوجيا يوميًا (مثل أجهزة الكمبيوتر، الهواتف المحمولة، والإنترنت، إلخ)؟
   - ............................. ساعة/اليوم
7. ما مدى راحتك في التعامل مع التكنولوجيا؟
   - □ غير مريح
   - □ مريح إلى حد ما
   - □ مريح
8. كيف تصف خبرتك في استخدام التكنولوجيا؟
   - □ مبتدئ (ليس لدي خبرة)
   - □ متوسط (لدي خبرة)
   - □ متقدم (محترف)

**القسم 2: المعرفة والتصورات للجراحة الروبوتية**

1. هل سمعت من قبل عن الجراحة الروبوتية؟
   - □ نعم
   - □ لا (انتقل إلى السؤال رقم 11)
2. إذا كانت الإجابة "نعم"، من أين سمعت عنها؟
   - □ الإنترنت ومواقع التواصل الاجتماعي
   - □ الطبيب
   - □ وسائل الإعلام (تلفزيون، إذاعة، إلخ)
   - □ مجلة
   - □ الأقارب أو الأصدقاء
   - □ خضعت أنا/أحد معارفي لجراحة روبوتية
   - □ غير ذلك (يرجى التحديد) ....................
3. ما الذي تفهمه من مصطلح "الجراحة الروبوتية"؟
   - □ الروبوت يجري الجراحة بينما الجراح يراقب
   - □ الجراح يتحكم في الأدوات الروبوتية بالكامل
   - □ الروبوت يتبع أوامر مبرمجة مسبقًا من الجراح
   - □ الجراح غير موجود في غرفة العمليات والروبوت يجري العملية وفق البرمجة
   - □ لا أعرف
4. أي من الخيارات التالية تتشابه أكثر مع الجراحة الروبوتية؟
   - □ الجراحة التقليدية المفتوحة (قطع كبير في الجلد)
   - □ جراحة المناظير (جراحة بفتحات فتحات صغيرة)
   - □ جراحة الليزر
   - □ لا أعرف
5. ما رأيك في مهارة الجراحين الذين يستخدمون الروبوت مقارنة بغيرهم؟
   - □ أكثر مهارة
   - □ متشابهة في المهارة
   - □ أقل مهارة
6. ما رأيك في المستشفيات التي تستخدم الروبوت؟
   - □ أفضل من المستشفيات التي لا تستخدم الروبوت
   - □ مشابهة للمستشفيات التي لا تستخدم الروبوت
   - □ أسوأ من المستشفيات التي لا تستخدم الروبوت
7. هل تعتقد أن الجراحة الروبوتية ستحل محل العمليات الجراحية التقليدية؟
   - □ نعم
   - □ لا
   - □ لست متأكدًا
8. هل تعتقد أن استخدام الروبوت في العمليات يمكن أن يحسن النتائج الجراحية؟
   - □ نعم
   - □ لا
   - □ لست متأكدًا

**القسم 3: تقييم التجربة الشخصية مع الجراحة الروبوتية**

1. ما نوع الجراحة الروبوتية التي خضعت لها؟
   - □ جراحة استئصال البروستاتا الروبوتية
   - □ جراحة استئصال الرحم الروبوتية
   - □ جراحة الكلى الروبوتية
   - □ جراحة المرارة الروبوتية
   - □ غير ذلك (يرجى التحديد) ....................
2. لماذا اخترت الجراحة الروبوتية؟ (اختر السبب الرئيسي):
   - □ أكثر أمانًا
   - □ أقل ألمًا
   - □ أسرع
   - □ الجراح أوصى بها
   - □ غير ذلك (يرجى التحديد) ....................
3. بشكل عام، كيف كانت تجربتك مع الجراحة الروبوتية؟
   - □ ممتازة
   - □ جيدة جدًا
   - □ جيدة
   - □ سيئة
   - □ سيئة جدًا
4. كيف كان مستوى الألم لديك في الأسبوع الأول بعد الجراحة الروبوتية؟
   - □ لا يوجد ألم
   - □ ألم خفيف
   - □ ألم متوسط
   - □ ألم شديد
   - □ ألم شديد جدا
5. كيف كان وقت التعافي من الجراحة الروبوتية مقارنة بوقت التعافي من العمليات الجراحية الأخرى (إن أمكن)؟
   - □ أسرع بكثير
   - □ أسرع
   - □ نفس المدة تقريبا
   - □ أبطأ
   - □ أبطأ بكثير
6. متى تمكنت من استئناف انشطتك العادية بعد الجراحة الروبوتية؟
   - □ فورًا (في خلال يوم)
   - □ في خلال أيام قليلة
   - □ في خلال أسبوع
   - □ في خلال شهر
   - □ أكثر من شهر
7. ما تقييمك لدقة الجراحة الروبوتية من حيث تقليل حجم الجرح والندوب؟
   - □ ممتازة
   - □ جيدة جدًا
   - □ جيدة
   - □ سيئة
   - □ سيئة جدًا
8. ما تقييمك لفعالية الجراحة الروبوتية من حيث حل مشكلتك الطبية؟
   - □ فعالة جدا
   - □ فعالة
   - □ غير فعالة
9. هل واجهت أي مضاعفات أثناء الجراحة الروبوتية أو بعدها؟
   - □ لا مضاعفات
   - □ مضاعفات طفيفة
   - □ مضاعفات متوسطة
   - □ مضاعفات خطيرة
   - □ مضاعفات تهدد الحياة
10. هل تنصح الآخرين بإجراء الجراحة الروبوتية إذا احتاجوا إليها؟
    - □ نعم
    - □ لا
    - □ لست متأكدًا

**English version of RAS patient questionnaire**

**Section 1: Demographics and Technology Experience**

1. **Name**
   ______________________________________________________
2. **Age**
   ______________________________________________________ years
3. **Gender**:
   - ⬜ Male
   - ⬜ Female
4. **Nationality**:
   - ⬜ Egyptian
   - ⬜ Non-Egyptian
5. **Education Level**:
   - ⬜ Primary
   - ⬜ Preparatory
   - ⬜ Secondary
   - ⬜ Diploma
   - ⬜ University
   - ⬜ Postgraduate
6. **On average, how many hours per day do you use technology (e.g., computers, mobile phones, internet, etc.)?**
   ____________________________ hours/day
7. **How comfortable are you with using technology?**
   - ⬜ Not comfortable
   - ⬜ Somewhat comfortable
   - ⬜ Comfortable
8. **How would you describe your experience with technology?**
   - ⬜ Beginner (no experience)
   - ⬜ Intermediate (some experience)
   - ⬜ Advanced (professional)

**Section 2: Knowledge and Perceptions of Robotic Surgery**

1. **Have you ever heard of robotic surgery?**
   - ⬜ Yes
   - ⬜ No (skip to Question 11)
2. **If "Yes," where did you hear about it?**
   - ⬜ Internet/Social Media
   - ⬜ Doctor
   - ⬜ Media (TV, radio, etc.)
   - ⬜ Magazine
   - ⬜ Relatives/Friends
   - ⬜ I/a relative underwent robotic surgery
   - ⬜ Other (please specify) ____________________________
3. **What do you understand by the term "robotic surgery"?**
   - ⬜ The robot performs the surgery while the surgeon observes
   - ⬜ The surgeon fully controls robotic tools
   - ⬜ The robot follows pre-programmed commands from the surgeon
   - ⬜ The surgeon is absent; the robot operates autonomously
   - ⬜ I don’t know
4. **Which of the following is most similar to robotic surgery?**
   - ⬜ Traditional open surgery (large incision)
   - ⬜ Laparoscopic surgery (minimally invasive with small incisions)
   - ⬜ Laser surgery
   - ⬜ I don’t know
5. **What is your opinion about the skill of surgeons who use robots compared to others?**
   - ⬜ More skilled
   - ⬜ Equally skilled
   - ⬜ Less skilled
6. **What is your opinion of hospitals that use robotic surgery?**
   - ⬜ Better than hospitals without robots
   - ⬜ Similar to hospitals without robots
   - ⬜ Worse than hospitals without robots
7. **Do you think robotic surgery will replace traditional surgery?**
   - ⬜ Yes
   - ⬜ No
   - ⬜ I’m not sure
8. **Do you believe robotic surgery can improve surgical outcomes?**
   - ⬜ Yes
   - ⬜ No
   - ⬜ I’m not sure

**Section 3: Evaluation of Personal Experience with Robotic Surgery**

1. **What type of robotic surgery did you undergo?**
   - ⬜ Robotic prostatectomy
   - ⬜ Robotic hysterectomy
   - ⬜ Robotic kidney surgery
   - ⬜ Robotic gallbladder surgery
   - ⬜ Other (please specify) ____________________________
2. **Why did you choose robotic surgery? (Select the main reason)**
   - ⬜ Safer
   - ⬜ Less pain
   - ⬜ Faster recovery
   - ⬜ Recommended by the surgeon
   - ⬜ Other (please specify) ____________________________
3. **Overall, how was your experience with robotic surgery?**
   - ⬜ Excellent
   - ⬜ Very good
   - ⬜ Good
   - ⬜ Poor
   - ⬜ Very poor
4. **How was your pain level in the first week after robotic surgery?**
   - ⬜ No pain
   - ⬜ Mild pain
   - ⬜ Moderate pain
   - ⬜ Severe pain
   - ⬜ Very severe pain
5. **How was the recovery time compared to other surgeries (if applicable)?**
   - ⬜ Much faster
   - ⬜ Faster
   - ⬜ About the same
   - ⬜ Slower
   - ⬜ Much slower
6. **When were you able to resume normal activities after robotic surgery?**
   - ⬜ Immediately (within a day)
   - ⬜ Within a few days
   - ⬜ Within a week
   - ⬜ Within a month
   - ⬜ More than a month
7. **How do you rate the precision of robotic surgery in minimizing scars/wound size?**
   - ⬜ Excellent
   - ⬜ Very good
   - ⬜ Good
   - ⬜ Poor
   - ⬜ Very poor
8. **How effective was robotic surgery in addressing your medical issue?**
   - ⬜ Very effective
   - ⬜ Effective
   - ⬜ Ineffective
9. **Did you experience any complications during or after robotic surgery?**
   - ⬜ No complications
   - ⬜ Minor complications
   - ⬜ Moderate complications
   - ⬜ Severe complications
   - ⬜ Life-threatening complications
10. **Would you recommend robotic surgery to others if needed?**
    - ⬜ Yes
    - ⬜ No
    - ⬜ I’m not sure
