# Supplementary material for: Robotic-assisted surgery in Egypt: national insights into awareness, knowledge, and perceptions among surgeons and patients
Source: J Robot Surg. 2025 Nov 21;20(1):32. doi: 10.1007/s11701-025-02942-w (PMC12634703; doi:10.1007/s11701-025-02942-w)
Supplement: Supplementary file 4 — Supplementary Material 4 [file 11701_2025_2942_MOESM4_ESM.docx]

**Journal of Robotic Surgery**

**“Tables and figures”**

**Robotic-Assisted Surgery in Egypt: National Insights into Awareness, Knowledge, and Perceptions Among Surgeons and Patients**

Mohamed F. Srour^1^, Ahmed H. Shoaib^2^, Hazim Alkousheh^3^, Karim K. Eladawy^4^, Mohamed Alayat^5^, Ahmed Abdelhameed^5^, Osama Alhaddad^4^, Seif M. Elsadik^6^, Ezzeldin Ahmed Abdelaty^7^, Mohamed Sloma^5^, Nada Rady^6^, Mohammad A. Abd-erRazik^8^

*Affiliations*

1. Faculty of Medicine, Menoufia University, Menoufia, Egypt.

ORCID: 0009-0008-4305-1971

1. Faculty of Medicine, Alexandria University, Alexandria, Egypt.

ORCID: 0009-0005-0708-6569

1. Faculty of Medicine, The Hashemite University, Zarqa, Jordan.

ORCID: 0009-0003-7175-7529

1. Faculty of Medicine, Ain Shams University, Cairo, Egypt.

ORCID (K.K.E): 0009-0002-9032-7017

ORCID (O.A): 0009-0003-7454-1838

1. Faculty of Medicine, Al Azhar University, Cairo, Egypt.

ORCID (M.A): 0009-0008-0183-6869

ORCID (A.A): 0009-0006-5491-1143

ORCID (M.S): 0009-0001-8998-918

1. Faculty of Medicine, 6th of October University, Giza, Egypt

ORCID (S.M.E): 0009-0007-1362-9923

ORCID (N.R): 0009-0003-5788-9082

1. Faculty of Medicine, Al-Azhar University, Damietta, Egypt.

ORCID: 0009-0004-4552-6919

1. General Surgery, Faculty of Medicine, Ain Shams University, Cairo, Egypt.

ORCID: 0000-0002-8498-9957

*Correspondence*

Mohamed F. Srour, MBBCh

Faculty of Medicine, Menoufia University

Address: Menoufia - Egypt

Phone Number: +201279012239

E-mail: [mohamed.ft.srour@gmail.com](mailto:mohamed.ft.srour@gmail.com)

**Index**

| **Supplementary Content:** | | |
| --- | --- | --- |
| **a. Tables:** | | **Page** |
| **1** | **Table 1:** Multivariable Predictors of Surgeon Support for Introducing Robotic-Assisted Surgery into the Healthcare System | **3** |
| **b. Figures:** | | **Page** |
| **1** | **Figure 1:** Patients’ source of hearing about RAS | **4** |
| **2** | **Figure 2:** Surgeon’s source of hearing about RAS | **4** |
| **3** | **Figure 3:** Reasons for surgeons’ opinions about introducing RAS in the healthcare system | **5** |
| **4** | **Figure 4:** Surgeons’ opinions about the overall cost of RAS compared to laparoscopic surgery | **5** |

**Table 6:** Multivariable Predictors of Surgeon Support for Introducing Robotic-Assisted Surgery into the Healthcare System

| **Predictor** | **Adjusted Odds Ratio (aOR)** | **95% Confidence Interval** | **p-value** |
| --- | --- | --- | --- |
| Age (per year) | 0.96 | 0.93 – 0.99 | 0.013 |
| Gender | 2.25 | 1.16 – 4.36 | 0.016 |
| Overall cost of RAS vs. laparoscopic surgery | 2.28 | 1.14 – 4.60 | 0.021 |
| Effect of RAS on recovery time | 2.93 | 1.14 – 7.49 | 0.025 |
| Understanding of robotic surgery | 2.49 | 1.48 – 4.18 | <0.001 |
| Availability of RAS in Egypt | 6.35 | 3.25 – 12.42 | <0.001 |

**CI = confidence interval**

Multivariable logistic regression analysis was performed to identify independent predictors of surgeon support for introducing robotic-assisted surgery (RAS) into the healthcare system. Adjusted Odds Ratio (aOR) greater than 1 indicates increased likelihood of support, while an aOR less than 1 indicates a decreased likelihood. Significant predictors included younger age, gender, perception of overall cost, perceived effect on recovery time, understanding of robotic surgery, and awareness of RAS availability in Egypt.


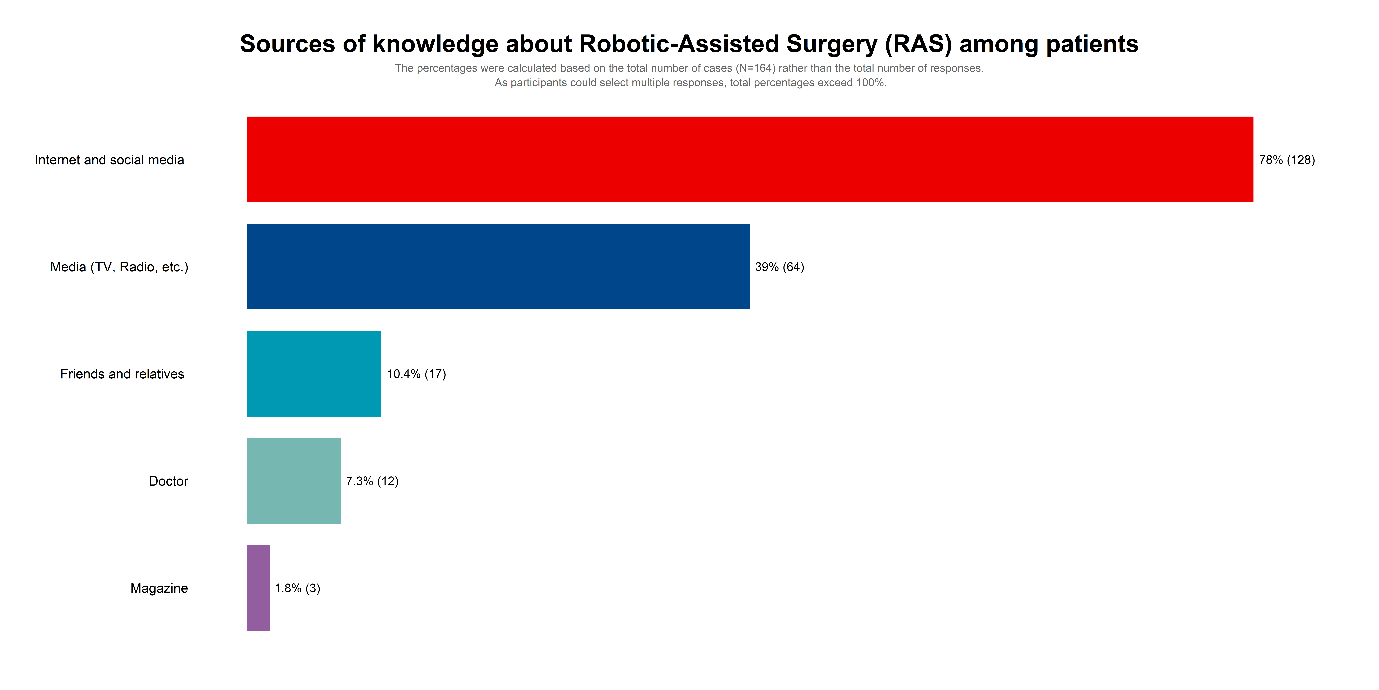


Figure 1: Patients’ source of hearing about RAS


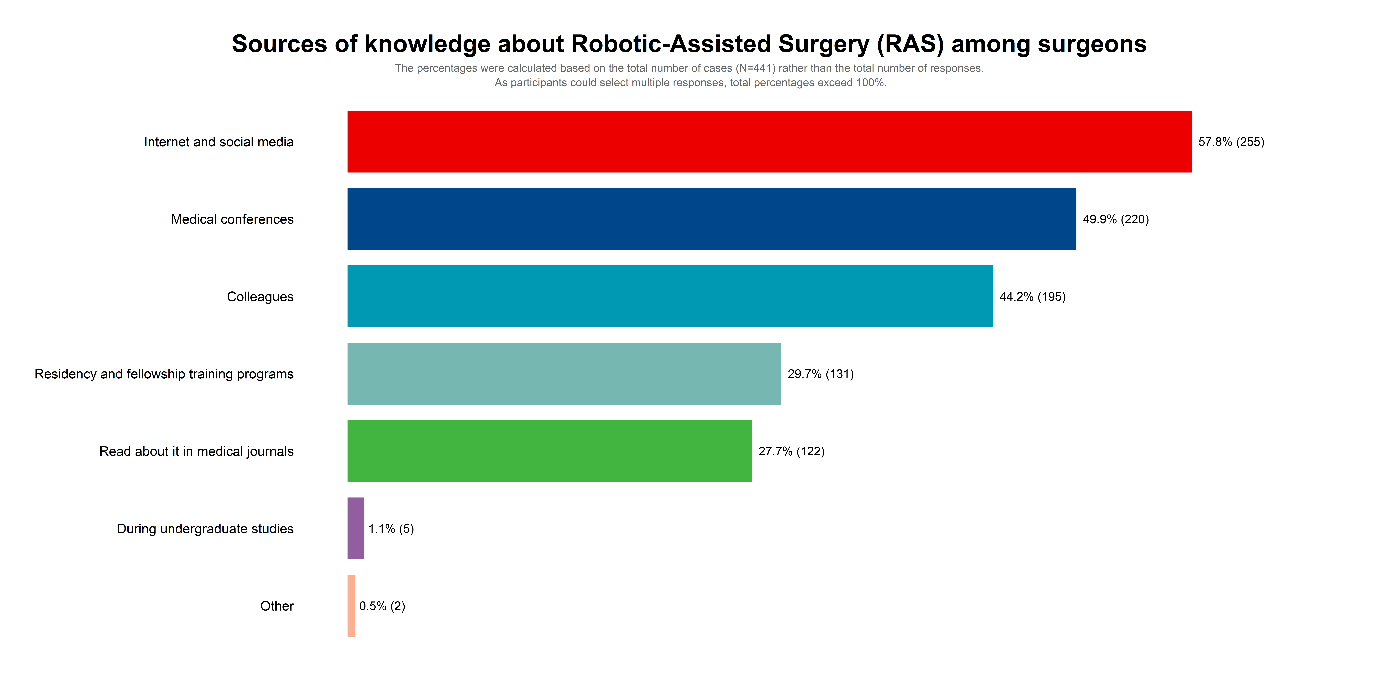


Figure 2: Surgeon’s source of hearing about RAS


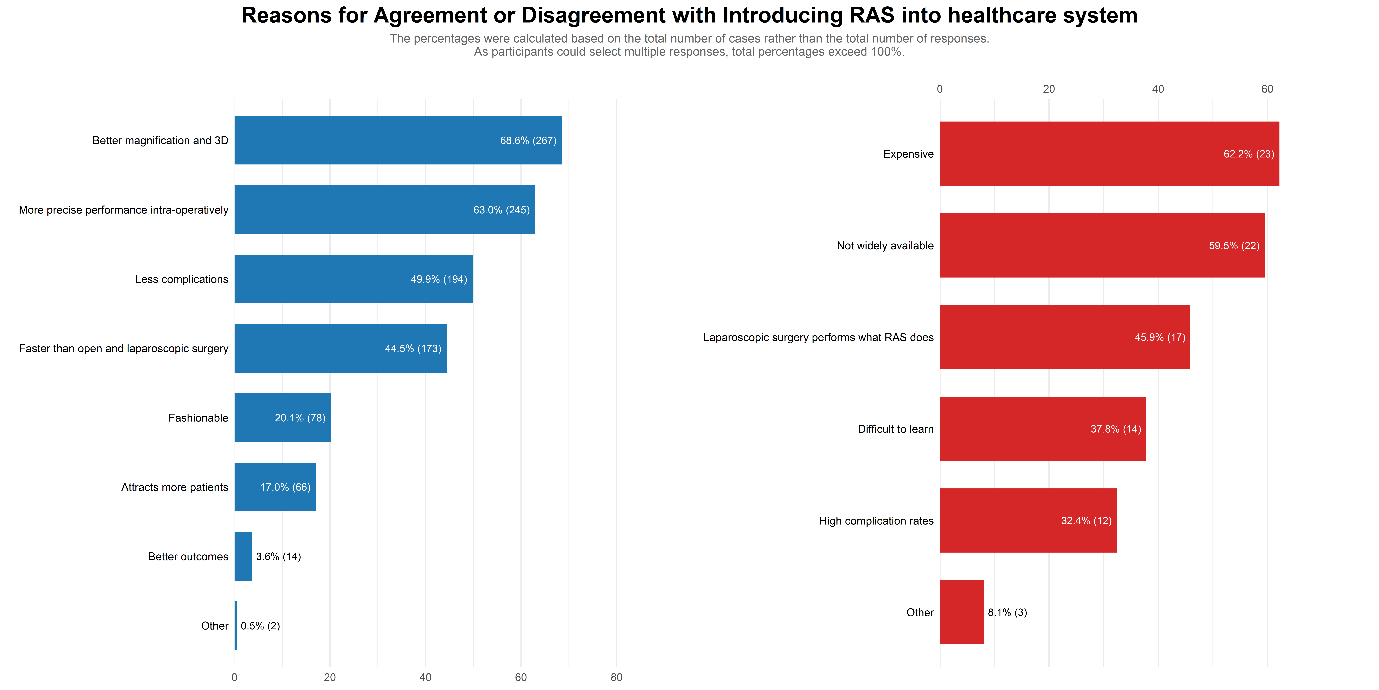


Figure 3: Reasons for surgeons’ opinions about introducing RAS in the healthcare system


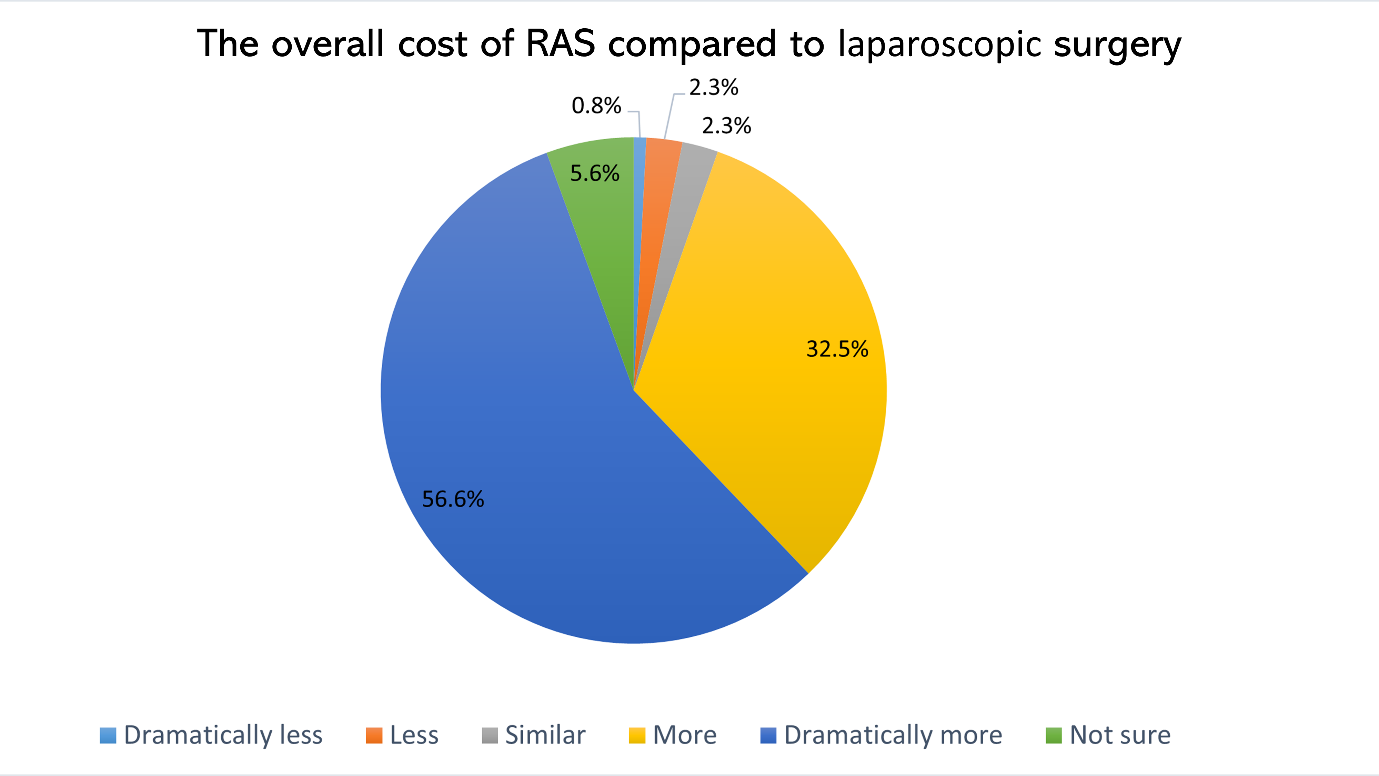


Figure 4: Surgeons’ opinions about the overall cost of RAS compared to laparoscopic surgery
